# Utilization Behavior: What Is Known and What Has to Be Known?

**DOI:** 10.1155/2014/297128

**Published:** 2014-02-09

**Authors:** Leonardo Iaccarino, Sergio Chieffi, Alessandro Iavarone

**Affiliations:** ^1^Vita-Salute San Raffaele University, Via Olgettina, 58, 20132 Milan, Italy; ^2^Department of Nuclear Medicine, San Raffaele Hospital, Via Olgettina, 58, 20132 Milan, Italy; ^3^Department of Experimental Medicine, Second University of Naples, Via Santa Maria di Costantinopoli, 16, 80138 Naples, Italy; ^4^Neurological and Stroke Unit, CTO Hospital, AORN, “Ospedali dei Colli”, Viale Colli Aminei, 21, 80131 Naples, Italy

## Abstract

Since the first description by Lhermitte (1983), the utilization behavior (UB) still represents an enigma for behavioral neurology and neuropsychology. Recent findings shed some light on new frameworks for interpreting this interesting phenomenon. Functional neuroanatomical basis is still unclear, although recent advances in neuroimaging techniques have contributed to a better understanding of the syndrome. An important and promising step is given by shifting researcher's attention from frontoparietal to intrafrontal mechanisms. From a cognitive standpoint, three models have been proposed. However, a comprehensive account for the UB neurobehavioral complexity is still lacking. Aims of this paper are to briefly review the reported cases of utilization behavior (UB) and to describe the putative neurological mechanisms underlying UB. Furthermore, the cognitive models proposed to interpret UB will be summarized. For clinical purposes, features suitable for distinguishing UB from other neurobehavioral symptoms will be briefly described.

## 1. Introduction

The utilization behavior (UB), starting from 1983 (year in which it was defined by the French neurologist Lhermitte [1]), has increasingly been a topic of interest, recently stimulated by the progresses of functional neuroimaging techniques. As Assal said about the Environmental Dependency Syndrome (EDS) [2]: “L'intérêt majeur de ce syndrome est qu'il touche directment au concept d'autonomie et à ce que l'on appelle le libre arbitre” (The major interest of this syndrome is that it directly implies the concept of autonomy and what we call “free will”). We can consider UB as the disorder in which the patient is forced to use or manipulate objects presented in a given context. What is surprising about the UB is that the patient does not detect any discrepancy between his actions and his intentions, so he will claim that “he wanted to do that.” The UB is often concomitant with another frontal sign, the imitation behavior (IB) and, together, they are the core symptoms of the EDS, that is, a more complex kind of dependence in which is the whole context that elicits scripts of behavior from the patient (see paragraph §3 for further details). Actually, there are still a lot of controversies about the UB, particularly referring to its neuroanatomical basis, its cognitive accounts, and its clinical value for both neurological (e.g., FTD onset [3, 4]) and psychiatric disorders (e.g., UB and attention deficit with hyperactivity disorder (ADHD) relationships [5, 6]). Only recent studies [7, 8] reported systematic neuroimaging evaluation about the individual differences of the UB phenomenology, with still unclear results. To date, three eliciting methods have been proposed to verify the presence of UB. The first method, devised by Lhermitte himself [1], simply consists in putting an object in the hands of the subject and then observing his behavior (assuming that “healthy” would ask something like what should I do? or why do you give me this?). The resulting utilization behavior is called “induced UB” [9]. Shallice et al. proposed a second method some years later [9], finding its “*raison d'être*” in a critical discussion of the Lhermitte approach. In this procedure, the UB is elicited by positioning an object on the desk, suddenly and not in front of the patient, in a way that should not let him think that he has to use it. If the object captures patient's attention and utilization occurs, this is the case of “incidental UB.” The third (and most recent) method has been proposed by Besnard et al. [7, 8]. This is called “verbal generation procedure” and is devised in the framework of the “embodied cognition” hypothesis [10]. Patients are asked to describe actions referring to some activities of daily living. While subjects are describing such activities, the examiner let some objects suddenly appear in front of the patient (by taking off a covering table/curtain). These objects may be related to the action (condition VG2) or not (condition VG1). The authors claim that their method can elicit UB as resulting from a “double activation.”

The UB has been associated with lesions of different brain areas [1, 11–15] as if with various (and often very different) conditions, from neurodegenerative diseases [3, 4, 16] to neuropsychiatric disorders [5, 6, 17].

Aims of this paper are (1) to briefly review all the cases in the literature in which UB has been identified as typical sign of both a neuropsychiatric disorder and a neurodegenerative disease; (2) to give a synopsis of the brain areas whose lesion has been reported as related to UB (see Table 1); (3) to describe the putative neurological mechanisms underlying UB; (4) to briefly describe the three main cognitive models proposed to explain UB [9, 18, 19]; and (5) to describe, for the purpose of clinical diagnosis, the differences between UB and other similar signs. Finally, limits of the studies as well as future directions in this research field will be highlighted.

## 2. The UB in Major Depression, ADHD, and FTD

The UB has been reported in various disorders, with relevant implications for the clinical practice, since the seminal study by Lhermitte [17]. The author investigated UB in a sample of 60 patients from a psychiatric ward. Eighteen of them, affected from major depression, showed IB or UB, sometimes even both. Lhermitte concluded that “the data show […] an unexpected focal specific neurological sign in a psychiatric disease.”

Recently, the ADHD has been associated with UB [5, 6]. In Nicpon et al.'s study, the authors verified Barkley's hypothesis [20] of frontal impairment as a neural basis of ADHD (assuming the UB as a “frontal sign”) by comparing boys with ADHD with matched normal controls. Subjects in the ADHD group exhibited more utilization behaviors and did so more quickly than boys in the control group; engagement with utilitarian objects and tendencies to do so quickly best predicted ADHD and control group membership. In the Archibald et al. study, the hypothesis of a frontostriatal involvement in the genesis of ADHD has been tested. Two raters recorded all the movement instances and the utilization behaviors of the subjects. UB was related to the severity of ADHD core symptoms, as well as to the visibility and familiarity of the object. The authors concluded that hyperactivity could be interpreted, at least in part, as UB.

Other interesting relationships have been highlighted, in two recent studies [3, 4] between the UB and the fronto-temporal dementia (FTD). In the former study, the UB was reported in 80% of patients with FTD, in contrast with 0% of subjects suffering from Alzheimer's disease (AD). These results were quite different from those by Bathgate et al. [21], who never observed UB neither in the FTD or AD groups. This divergence could be accounted, at least in part, to the different demographic and clinical characteristics of the patients under investigation. It is noteworthy that Ghosh et al.'s subjects had older mean age (59 years versus 62), shorter duration of the illness (mean years 2.5 versus 4.2), and lower mini mental state examination (MMSE) scores (mean 17 versus 21).

In the latter study [4], the authors showed that careful evaluation of environmental dependency behaviors, considered pathognomonic sign of frontal lesion, could clearly differentiate the behavioral variant of FTD from probable AD. In particular, UB (78% overall UB; 66% incidental UB) and IB (59%) occurred exclusively in behavioral variant of FTD (bvFTD). It is noticeable that authors collected data by reviewing personal history and observing spontaneous behavior by means of the known eliciting procedure. It should be stressed that UB is considered a typical positive symptom for the clinical diagnosis of bvFTD (e.g. [22, 23]). Furthermore, UB is included, in the positive subscale, in the most widely used inventory to assess frontal behavioral symptoms [24], which has shown the higher discriminating properties to differentiate FTD from other types of dementia [25–27]. Further evidence is provided by Lagarde et al. [16], who reported UB in two patients suffering from bvFTD.

## 3. The Heterogeneity of Site and Type of Brain Lesions Associated with UB

It is well known that the UB may be due to lesions of brain regions that are heterogeneous both for their site and nature [28]. The lesions reported originally by Lhermitte in his landmark paper [1] were very heterogeneous, ranging from “anterior right cortical-subcortical frontal lobe” to “ascending frontal gyrus.” The author also reported cases with caudate lesions, which have been as well described many years later [11, 29, 30]. A predominant cluster of right lesions was shown, arising from various etiological mechanisms. Together with the case reports, Lhermitte explained the eliciting method: “The objects are shifted within the field of vision, far away from the patient's hands, which incites the patient to make a large gesture with one of his upper limbs in order to grasp them. The hands of the patient being free, the examiner then shows a utilitarian object—a glass, for instance—within the field of vision of the patient, which he then brings within reach of one of the patient's hands. […]” [1].

An early study by Assal [2] presented a peculiar kind of environmental dependence upon written language. The 37-year-old patient read aloud all written words around her, from card to magazines. This could be classified as a case of hyperphasia [36]. Still, Lhermitte and coworkers, three years later, presented two other studies in which the UB was described in detail [11, 12]. In the former, the UB and IB are investigated as entities subtended by a common link. In particular, the UB is considered a further decline of previous impairment leading to the IB. The UB is defined as a “release of parietal lobe activities, resulting from impairment of frontal lobe inhibition.” In the latter, study the label of “environmental dependency syndrome” is coined for the first time. According to Lhermitte's definition, it has to be considered as a “disorder in personal autonomy.” The two patients described in this study showed a very peculiar behavior; for instance, the description of Lhermitte's house as a museum was enough to let them stare at the furniture and comment the sight of each picture.

An important study by Shallice et al. [9] addressed two important controversies about the UB. First, the authors suggest the distinction between an “induced UB (elicited by Lhermitte method)” and an “incidental UB.” exhibited by the patients while they are paying attention to another task. Furthermore, the utilization is classified into three categories, depending on the kind of utilization: toying, complex toying, and coherent activity. This distinction has been widely accepted [40] and compared with a new elicitation procedure [7]. Second, the authors propose a new cognitive frame accounting for the UB. Conversely to Lhermitte, who interpreted UB with reference to the historical model of Denny-Brown [41], Shallice et al. interpreted this behavior in the framework of the Supervisory-Attentional System (SAS) model by Norman and Shallice [18]. Within the SAS, the UB may be looked as resulting from the loss of a “working supervisory system”, in which the frontal lobes play the main role. For instance, in a patient with UB, the simple sight of a trigger stimulus (e.g., scissors) will be enough to activate the related schemata; as consequence, the subject will act the related behavior, not inhibited by control systems (see paragraph §5 for further details).

In 1991, Eslinger et al. described a patient with a paramedian thalamic lesion following infarction [13]. This study has contributed to shift interpretation of anatomical basis of UB from “fronto-parietal to intrafrontal mechanisms” [42]. In 1992, Hoffmann and Bill reported a new case of EDS due to the moyamoya disease [31]. The patient, brought to a lecture room, with a board and pens available, soon behaved as the teacher (that was his former job), considering the doctors as his pupils. This is also the first case reported in which the IB, UB, and EDS are caused by a bilateral prefrontal damage. The second case of UB due to a bilateral frontal damage was described, a few months later, by Fukui et al. [32]. This represents the first case in which the UB, termed by the authors “manual grasping behavior” (MGB), was associated with motor neglect, probably caused by a lesion of the right supplementary motor area (SMA). Another patient, described by Degos et al. [29], presented UB in the context of a “severe frontal syndrome,” caused by anterior cingulate and caudate lesions. A relevant contribution to the study of UB and EDS was given in the following years by Brazzelli et al. [33, 34], highlighting intriguing patterns of spared and impaired cognitive abilities. P.G. was a 16-year-old girl who had suffered from herpetic encephalitis. She presented bilateral lesion of frontal orbitomesial areas with involvement of the cingulate cortex. She showed also “psychotic-like” spontaneous behavior, for example, speaking sometimes in *falsetto* voice. The incidental UB presented by P.G. was found by the authors to be coherent with SAS impairment [18]. In a relevant study by Ghika and coworkers, the UB was investigated in the context of a progressive supranuclear palsy (PSP) [35]. In this disease, along with other Parkinsonian-spectrum disorders, the impairment of frontostriatal pathways (in particular descending traits) led to a “chain of behaviors.” Another interesting report is by Tanaka et al. [36], about an old woman who suffered from left frontal infarction and was admitted to the hospital with forced grasp reflex, IB, and gait disturbance. Even though IB regressed by two weeks, more intriguing behaviors appeared “[…] In the presence of others, she would call out the names of objects in the room and also call out the actions and gestures of people in the room […]”. The authors hypothesized an impairment of the frontal inhibitory function. The patient C.U. presented by Boccardi et al. [37] is the first case reported in which UB has been described following a bilateral SMA lesion (caused by a stroke). The authors claim that the UB could be conceived as a “double anarchic hand,” due to an imbalance “between the premotor cortices, responsive to environmental triggers, and the supplementary motor areas, which modulate actions and inhibit them.” This study supports the already mentioned “intrafrontal hypothesis” about the anatomical basis of UB pointed out by Eslinger [42]. Ishihara et al. [14] presented a case of a neuropathologically verified 72-year-old patient who, at admission, showed incidental and induced UB. He suffered from infarction of the subcortical white matter involving the superior left frontal lobe. This finding led the authors to hypothesize that UB could be considered a “white matter disconnection syndrome.” A particular (and somewhat spectacular) case of EDS-form has been recently described by Conchiglia et al. [38] and termed “Zelig-like Syndrome.” The patient (A.D.) is a 65-year-old politician (and amateur actor) who suffered from cerebral hypoxia (with frontotemporal lesions) due to a cardiac arrest. The authors report that “[…] he assumed a different social role in keeping with different environmental circumstances by interpreting a character corresponding to the particular context […]”. They interpret the syndrome as a loss of frontal inhibition, whose function is the control of his own identity; as consequence, the subject exhibits “attraction” towards a social role proposed by the environment. Important contributions to methodological and theoretical issues about UB come from two recent studies by Besnard et al. [7, 8]. In the first study, the authors propose a new elicitation method, called “verbal generation procedure”; claiming that it would be more accurate to elicit the symptom as based on a “doubl-activation.” In addition, they highlight that the efficacy of their procedure is more associated with the results of the induced approach as compared with incidental one. Furthermore, in the attempt to explain the dissociation between UB and the normal performances at tasks of inhibitory control (e.g., Stroop test), the authors point to the role of social components, in particular a disorder involving Theory-of-Mind (ToM), which would better account for the observed symptoms. The “social hypotheses” have been further investigated in the second study, which involved 60 neurological patients with frontal (n. 30), subcortical (n. 20), and posterior (n. 10) brain lesions. All the three methods for eliciting UB were applied. The authors found no UB with the “incidental” method, but a frontal specificity was observed as a result of “induced” and “verbal generation” methods (3/30 and 12/30 patients resp.). The authors also propose a new cognitive frame accounting for the UB (see below). An interesting study by Spiegel and Lamm reports a case of hyperorality [30]. The lesions involved bilaterally the medial frontal lobes and the left caudate nucleus. The 29-year-old patient showed UB, bulimic-type eating and hyperorality, a form of dependence consisting in eating or bringing to the mouth everything edible or inedible that is in the sight of the subject. The last two symptoms are part of the core features of the Klüver-Bucy Syndrome [43], whose lesions classically involve bilaterally medial frontal and temporal lobes. The case described led authors to highlight the main role played by lesion of medial frontal areas in determining certain Klüver-Bucy symptoms, which can be associated with UB.

Recently, Balani et al. [39] described the case of F.K., a patient who had also been included in a sample of a study focused on action disorganization syndrome [44] (see paragraph §6 further for details on differential diagnosis). He suffered from carbon monoxide poisoning, resulting in lesions involving medial frontal and temporal areas, as well as cingulate cortex. The author's aim was to disentangle the roles of bottom-up and top-down mechanisms in the UB, starting from the hypothesis that the UB results from a bottom-up strategy involvement. The authors interpret their results as supporting the role of top-down mechanisms and hypothesize the existence of at least two components underlying UB, that is, (1) “a failure to prioritize “task-based goals” over other ongoing information held in Working Memory (WM)”; (2) “a problem in response inhibition once task-inappropriate information has been activated” [39].

## 4. Putative Neurological Mechanisms

It is not the purpose of a short review focused on UB to discuss the cortical control of movements. An important role is played by prefrontal and associative areas that match the motor commands with personal goals before the transduction by the motor cortices into movements. The original model by Denny-Brown and Chambers [41, 45], that has been then formalized by Mesulam in 1986 [46], is the basis of Lhermitte interpretation. The model assumes the existence of two competitive biological orientations (tropisms), an approaching/excitatory one, dependent upon more posterior cerebral systems, and a withdrawal/inhibitory one, based upon anterior systems. The hypothesis is that UB is given by impairment of this last mechanism, leading to a non-inhibition of the “chain of behaviors”. Another broadly accepted model is that proposed by Goldberg [47]. The author hypothesizes two different systems: medial and lateral. This distinction is based upon the differentiation of internally- and externally-guided actions. The medial system, including SMA and cingulate cortex, is related to the internally-driven actions; the effect of lesions of these areas would lead to a critical reduction of intended action (e.g. akinesia/mutism). As consequence, a dominance of externally-driven actions would be observed, as it happens in the course of UB (for a complete review, see Goldberg [47] or the detailed analysis by Archibald et al. [40]); see also comments by Eslinger [42].

## 5. Cognitive Models Proposed for the UB

### 5.1. The Supervisory-Attentional System (SAS) Model

The first cognitive model to interpret the UB (except the original model by Denny-Brown and Chambers [41, 45]) is the SAS model proposed by Norman and Shallice [18] after the seminal paper held in 1982 [48]. This model also represents the theoretical framework [18] to which refer several already cited contributions [6, 7, 33, 34, 38]. The SAS is a cognitive neuropsychological model that postulates the existence of four modules:

- Special-Purpose Cognitive subsystems,
- schemata,
- contention scheduling,
- Supervisory-Attentional System (in strict sense).

The first module includes the whole number of the specific subsystems related to the task; for example, in a reaching-and-grasping task, we need to perform object recognition, distance estimation and so on. Shallice defines the schemata as “analogous to programs that “run” on these cognitive subsystems.” In this perspective, we refer to reaching and grasping as schemata activated by the task. The Contention Scheduling is a “mechanism by which appropriate schemata are selected to control behavior, primarily through schemata being in a mutually inhibitory relation.” The SAS plays an important role in ambiguous situations, in which no schema is activated more than another and the contention scheduling cannot afford the requests of the context. This system can thus provide additional activation or inhibition of the schemata, so that a new situation can be managed with a proper strategy. According to the described model, UB can be considered as resulting from SAS impairment, due to a failure in monitoring schemata. Therefore, the more a trigger is relevant for the patient, the more a strong schema will be activated. As a consequence, it is highly probable to not observe UB when another schema is activated (e.g., when the patient is focusing on a different task).

### 5.2. A Motor Control System Based Upon Engineering Principles

Another cognitive perspective is that by Frith and coworkers [19] (for a complete review, see [49–51]). This model assumes that “a well-functioning motor system is an essential requirement if we are to move through our environment safely” [19]. Fundamental importance is assigned to sensory information and to the ability of rapidly detecting the consequences of actions in the environment. The brain is able to make prevision about the consequences on the musculoskeletal system of every motor command that is going to be executed. Still, other kinds of information are needed to make such prevision, like all the proprioceptive information about flexor angles and arm position in the space (which the authors call “state variables”). Given the role of all these variables (which represent aspects of the body or of the context [49–52]), Frith et al. postulate two main types of internal models, namely predictors (forward models) and controllers (inverse models). We refer to the predictors as the internal models that allow the estimation of sensory consequences of a motor command and to the controllers as the internal models that perform an analysis of the relation between the desired state and the movement “required to achieve that state”. The effective functioning of the two internal models will necessary imply the correct representation of the actual system state, the desired system state, and the predicted system state. At this purpose, it is important to keep in mind that a relevant feature of the UB is an impaired ability, by subjects, to detect a discrepancy between actions and intentions. Patients seem not being surprised by their utilizations, nor disappointed by their actions (see paragraph §6 for details on differences with Alien Hand Syndrome). The experience of perceiving an action as intentional is based on the congruency between the action and the personal goal. The UB is then the consequence of two dysfunctions: first, a lack of awareness about personal goals, so that the patient is not aware of the action until he has performed it; second, the inappropriate actions elicited by external affordance are not inhibited. In conclusion, the lesions underlying UB impair the ability to represent personal goals; therefore, when an action is elicited by environment, it is impossible to inhibit it because of the lack of a desired state to consider as a reference. The absence of goals, on the other hand, does not allow performing both appropriate (to select) and inappropriate (to inhibit) actions.

### 5.3. The Social Hypothesis

In their study of 2011 [8], Besnard et al. pointed to potential limitations about Shallice et al.'s and Frith et al.'s cognitive approach, since their models “provide no explanation for imitation behavior, the other side of environmental dependency.” Indeed, it is hard to interpret the IB as an “activation of overlearned actions by a trigger stimulus.” Recently, the IB has been related with the default-mode-network (DMN) [53], as well as to the mirror neuron system (MNS) [54], and it has been nonetheless associated with an executive deficit. With regard to IB-DMN association, the authors themselves make an explicit reference to the SAS model, while the relationship between IB and MNS or executive functioning could be subtended by several kinds of mechanisms [55]. As claimed by Besnard et al., IB and UB, although both linked to a frontal lobe syndrome, do not significantly correlate with scores of neuropsychological tests assessing executive functioning: “frontal patients who were “dependents” did not differ from “nondependents” for executive tasks, except for the time completion of the Stroop test” [8]. To explain this discrepancy, the authors suggest a new “social hypothesis”, based upon the existence of a complex social interaction between the patient and the examiner [56], stating that the “understanding of social situations is often automatic and immediate, allowing us to create a “shared world””[8]. Further support to this idea comes from recent studies [57, 58] and is also consistent with the hypothesis of Gallese [59] about the “social metacognition.” In this perspective, the representation of examiner's intentions by the patient should play a crucial role, thus supporting the hypothesis of a “strong relation between a theory of mind (ToM) disorder and the presence of environmental dependency” [8].

As final remark, we would stress that all the cognitive frameworks briefly described are far to be exhaustive for the UB phenomenon, as each one fails to give complete and comprehensive explanations for all the symptoms of the syndrome.

## 6. Differential Diagnosis

From the clinical standpoint, it is quite important to distinguish the UB from other kinds of pathological manipulation and grasping.

### 6.1. UB and ADS (Action Disorganization Syndrome)

Both the syndromes involve actions and movements [44, 60–62]. However, the patient with UB is able to perform all the necessary steps to accomplish or execute a complex action. For example, he is able to hang a picture on the wall (take the measures, take the hammer, and so on). Conversely, a patient suffering from ADS is unable to execute a complex behavior (also routine) showing a wide variety of errors, from “place substitution” (e.g., while preparing the coffee, the patient takes a spoon of butter instead of the coffee powder) to “step omission” while performing a step-by-step action (e.g., putting the pot without the water on the gas).

In brief, when a patient is unable to accomplish routine actions, most likely he is not showing UB.

### 6.2. UB and Alien Hand Syndrome

The difference between UB and Alien Hand Syndrome (AHS) [37, 63–66], sometimes called anarchic hand, is more subtle, but relatively easy to detect. The UB is not associated with rejection of the agency by the patient; in other words, he is somewhat “syntonic” with his own actions. On the contrary, the patient with AHS is aware of his disorder, often reporting: “the arm is moving by itself.” It is noteworthy that an “anarchic hand” may also perform complex movements, and it is not rare to observe the so-called “inter manual conflict,” in which the alien hand is opposing the movement performed by the other hand. An interesting contribution for understanding the difference between UB and AHS is the study by Pacherie [67], which compares two approaches to the agentive self-awareness: the holistic narrator-based approach and the atomistic comparator-based approach.

In brief, when the patient is telling you he did not want to perform the movements just executed, UB can be excluded.

### 6.3. UB and Grasping Reflex

The grasping reflex (GR, or palmar grasping reflex) as defined by Denny-Brown and Chambers in 1958 [45] is the automatic tendency of the infant (or the patient) to grasp an object. The reflex can be elicited by putting examiner's hand (or just one finger) in the hand of the patient. Sometimes, the patient is unable to release the grip (forced grasping). In literature we can find as synonym of GR also other terms, like Manual Grasping Behavior or Magnetic Apraxia.

Thus, a patient unable to stop in manipulating an object (e.g., during a Lhermitte elicitation procedure), in spite of the reinforcement by the examiner, is probably not affected by UB, although the cooccurrence of the UB and GR cannot be excluded.

### 6.4. UB and Manual Groping Behavior

Another reflex sometimes present in patients with frontal lobe damage is the Manual Groping Behavior (also referred to as groping reflex) [68–70]. In this case, the patient seems to be magnetically attracted by an object, and this phenomenon may happen for both manually and visually processed stimuli.

As claimed by Archibald et al. [40], the groping reflex is characterized by the fact that “the behaviors do not appear to be volitional or purposeful and are very repetitive and stereotypic.” This feature contributes to differentiating groping behavior from UB, in which movements are goal directed.

## 7. Future Directions

Future studies will address many of the unresolved questions about UB. (1) The need of a clear neuroanatomical model: as, since that already described by Goldberg [47], new comprehensive proposals are still lacking. These should take into account the new “intrafrontal” components recently highlighted [42]. (2) The need of connectivity studies: in fact, even though white matter impairment in the UB could be hypothesized [14], to date no studies have been carried on by means of the diffusion tensor imaging (DTI) in the “utilizers.” (3) In spite of the three different procedures to elicit UB, a clear taxonomy is still lacking. (4) From the cognitive standpoint, future contributions will aim to provide comprehensive models able to include different aspects of motor behavior, as if to predict the heterogeneous features of UB in terms of selective involvement of cognitive components. These models would take into account aspects belonging not only to “mechanical” features of the movement, but also dimensions belonging to social cognition and supervisory functions.

## Figures and Tables

**Table 1:** Summarizing data for lesion sites shown in literature to be concomitant with utilization behavior (UB) or Environmental Dependency Syndrome (EDS).

	Orbitofrontal region	Caudate nucleus	Mesial frontal lesions	Thalamus	Frontal white matter	Frontal and prefrontal cortex	Inferior frontal lobe	Superior frontal regions	Cingulate cortex (gyrus)	Temporo-polar lesion	Temporo-mesial lesion	Fronto-striatal projections	Supplementary motor area	Fronto-temporal lesions	Paraventricular and subventricular regions	Rolandic region	Olfactory cortex
Lhermitte, 1983 [1]	X	X															
Assal, 1985 [2]			XB				XR										
Lhermitte et al., 1986a [11]		XB	X	XL													
Lhermitte et al., 1986b (EDS) [11]					X		X										
Shallice et al., 1989 [9]			XB				XB										
Eslinger et al., 1991 [13]				XB													
Hoffmann and Bill, 1992 [31]						XB											
Degos et al., 1993 [29]		XR							XL								
Fukui et al., 1993 [32]									XB								
Brazzelli et al., 1994/1998 [33, 34]	XB		XB			XB			X	X	X		X				
Ghika et al., 1995 (EDS) [35]												X					
Hashimoto et al., 1995 [15]				XR													
Tanaka et al., 2000 (EDS) [36]								XL						XL			
Boccardi et al., 2002 [37]													XB				
Ishihara et al., 2002 [14]					XL			XL									
Conchiglia et al., 2007 (EDS) [38]														XL			
Ghosh and Dutt, 2010, 2012 [3, 4]														XB			
Besnard et al., 2010 [7]	X					X							X		X	X	
Spiegel and Lamm, 2011 (EDS) [30]		XL	X														
Besnard et al., 2011 [8]	X		X			X	X	X						X			X
Balani et al., 2012 [39]			XB						X		XB			XB			

X: involvement without specified laterality; XB: bilateral involvement; XL: left hemisphere involvement; XR: right hemisphere involvement.
